# New Polyphenols Identified in *Artemisiae abrotani herba* Extract

**DOI:** 10.3390/molecules200611063

**Published:** 2015-06-15

**Authors:** Elisabeta Baiceanu, Laurian Vlase, Andrei Baiceanu, Madalina Nanes, Dan Rusu, Gianina Crisan

**Affiliations:** 1 Pharmaceutical Botany Department, Faculty of Pharmacy, Iuliu Hațieganu University of Medicine and Pharmacy, 23 Marinescu Street, Cluj-Napoca 400037, Romania; E-Mails: comsa.elisabeta@umfcluj.ro (E.B.); gcrisan@umfcluj.ro (G.C.); 2 Pharmaceutical Technology and Biopharmaceutics Department, Faculty of Pharmacy, Iuliu Hațieganu University of Medicine and Pharmacy, 12 I. Creanga Street, Cluj-Napoca 400010, Romania; 3 Physical Chemistry Department, Faculty of Pharmacy, Iuliu Hațieganu University of Medicine and Pharmacy, 6 Louis Pasteur Street, Cluj-Napoca 400349, Romania; E-Mails: madalinananes@yahoo.com (M.N.); drusu@umfcluj.ro (D.R.)

**Keywords:** *Artemisia abrotanum* L., polyphenols, flavonoids, hydroxycinnamic derivatives, phenolic acids, natural antioxidants, bioactive compounds, healthy nutrition

## Abstract

*Artemisia abrotanum* L. (“southernwood”) belongs to the *Artemisia* genus and it is used in traditional medicine for the treatment of a variety of illnesses. Scarce data is available on the chemical composition of this medicinal plant, most research being focused on the quantitative and qualitative analyses of its essential oil. Our aim was to investigate the content and profile of polyphenols, flavonoids and hydroxycinnamic derivatives present in the *Artemisiae abrotani herba* extract. We conducted LC/MS analysis and we screened for 19 polyphenols, flavonoids and hydroxycinnamic derivatives. We determined the total content of these compounds and we screened for antioxidant activity. Most polyphenol acids, hydroxycinnamic derivatives and flavonoids were identified and quantified for the first time in this study. We found an original polyphenol distribution profile with high concentration of sinapic acid, rutin, quercetol, ferulic acid and patuletin. We measured the antioxidant activity, the ethanolic extract presenting a modest radical scavenging activity. The value of this study consists in its novelty as it adds new data on the chemical composition of *A. abrotanum* L. and it opens novel perspectives for medical and nutritional applications of this plant.

## 1. Introduction

In recent decades, we have witnessed an increase of chronic diseases and associated complications. New strategies are required to tackle this problem [1]. Plant-derived compounds have been used in medicinal therapies for centuries [2]. Contemporary pharmacotherapy includes a wide range of drugs of plant origin [3]. Despite drug discovery technology advances and a decrease in funding for product-based drug discovery, natural products from plants and other biological sources remain an rich source of new possible therapeutic agents [2].

*Artemisia* L. is a genus of small herbs and shrubs found in northern temperate regions. It belongs to the *Compositae* (*Asteraceae*) family, one of the most numerous plant groupings. A large number of members of the *Artemisia* genus are used as ornamental plants, or as medicinal and aromatic plants for their essential oils and active compounds [4,5]. They are mentioned in folk and modern medicine, in the cosmetic and pharmaceutical industry, and they received special attention for their content of artemisinins which are active molecules against malaria [6,7,8].

Research on *Artemisia* species is focused on their essential oil composition and artemisinin content. Artemisinin is one of the fastest-acting pharmaceutical agents used against malaria [9]. Recently, compounds belonging to this class are investigated for the treatment of other conditions ranging from cancers and inflammatory diseases to viral and parasite-related infections. It was proved that *Artemisia* sp. can have much broader health applications in different types of conditions. Further information regarding these conditions can be found elsewhere [6]. The traditionally prepared *Artemisia annua* formulation is used as an anti-malaria drug worldwide and has been shown to have a better effect than purified artemisinin. This may is probably due to the synergistic action of other compounds found in the medicinal plant and because the total herbal extract might contain other molecules that lead to a more bioactive complex [9].

*A. abrotanum* L. (“southernwood”) belongs to the *Artemisia* genus. It is used as an aromatic plant and in traditional medicine for the treatment of a variety of disorders, such as infections (or inflammatory diseases) of the upper respiratory tract [4]. Studies on the isolation and characterization of pharmacologically active constituents have focused on the essential oil, which contains several bioactive molecules that confer the plant its anti-inflammatory, expectorant, spasmolytic, antiseptic and antimicrobial activities [10,11]. The composition of the essential oil of *Artemisia abrotanum* L. from Romania was investigated for the first time by a team of researchers from the “Iuliu Hațieganu” University of Medicine and Pharmacy, Cluj-Napoca. It was shown that its main constituent is eucalyptol [12].

Very little is known about the other constituents, such as polyphenols, of *A. abrotanum* L. that could have a biological activity. Polyphenols abound in natural products and in human diet and their roles in preventing chronic degenerative diseases have been shown in previous studies [13,14,15]. Some can be considered promising candidate agents for treatment of some cancers, while others play a role in the prevention of cancer [16]. Estimations for the average daily intake of mixed flavonoids in the U.S.A. were in the range of 0.5 to 1 g. The documented biological effects of dietary flavonoids include anti-inflammatory, anti-allergic, antimicrobial, hepatoprotective, antiviral, antithrombotic, cardioprotective, capillary strengthening, antidiabetic, anticarcinogenic and antineoplastic effects [15,17,18,19,20].

Our aim is to analyze the polyphenolic profile of the *Artemisiae abrotani herba* extract. The findings could lead to new therapeutic applications for *A. abrotanum* L.

## 2. Results and Discussion

### 2.1. LC/MS Polyphenol Analysis

**Figure 1:**
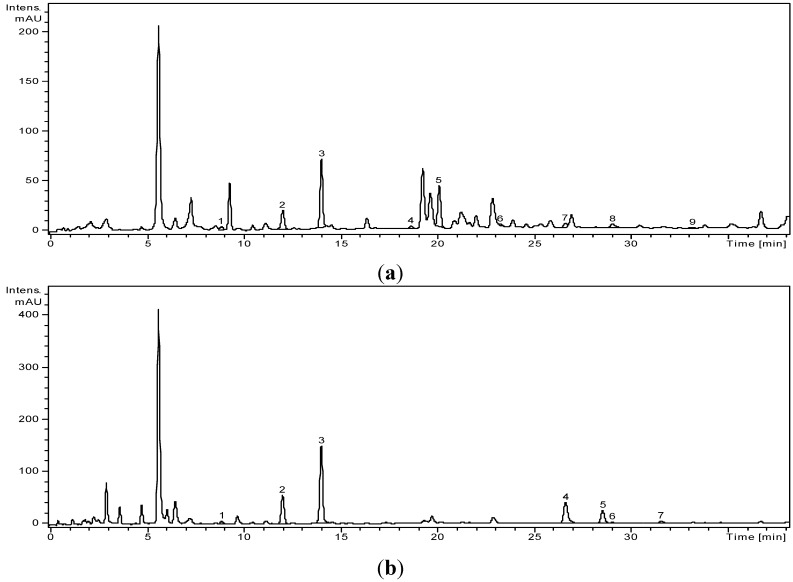
LC-MS profile of *A. abrotanum* (**a**) Before hydrolysis and (**b**) After hydrolysis. (**a**) *Artemisiae abrotani herba* extract HPLC chromatogram, before hydrolysis: 1. *p*-coumaric acid; 2. ferulic acid; 3. sinapic acid; 4. hyperoside; 5. rutoside; 6. quercitrin; 7. quercetol; 8. luteolin; 9. apigenin; (**b**) *Artemisiae abrotani herba* extract HPLC chromatogram, after hydrolysis: 1. *p*-coumaric acid acid; 2. ferulic acid; 3. sinapic acid; 4. quercetol; 5. patuletin; 6. luteolin; 7. kaemferol.

The high performance liquid chromatography (HPLC) assisted by mass spectrometry detection (MS) is a technique that suits well the requirements of the polyphenol screening in herbal extracts as it brings both high sensitivity and structural data on the identified biomolecules. The results of the qualitative and quantitative analyses of the polyphenol profile of *A. abrotanum* L. extracts (before and after hydrolysis) can be seen detailed in Figure 1.

Next, we analyzed the distribution of polyphenols in the extracts. We looked at the most common polyphenols found in plants from the same family and genus. We compared distributions both prior and post hydrolysis to see the differences in the polyphenol profile before and after the separation of the aglycone. The original distribution of polyphenols in the ethanolic extracts of *A. abrotanum* L. can be found in Table 1 and Table 2.

**Table 1:** Polyphenol profile of *Artemisiae abrotani herba* before hydrolysis.

Compound	Retention Time (min)	UV Detection	MS Detection	Concentration (µg∙mL^−1^)
Gentisic acid	2.15	NO	YES	NF
Caffeic acid	5.6	NO	YES	NF
Chlorogenic acid	5.6	NO	YES	NF
*p*-cumaric acid	8.7	YES	YES	2
Ferulic acid	12.2	YES	YES	10.31
Sinapic acid	14.3	YES	YES	34.56
Hyperozid	18.6	YES	YES	1.95
Isoquercitrin	19.6	YES	YES	NF
Rutin	20.2	YES	YES	62.9
Quercitrin	23	YES	YES	2.42
Quercetol	26.8	YES	YES	2.59
Luteolin	29.1	YES	YES	4.27
Apigenin	33.1	YES	YES	1.16

Notes: NF-not found, below limit of detection; Values are the mean ± SD (0.2%–1.5%) (*n* = 3); SD = standard deviation.

**Table 2:** Polyphenol profile of *Artemisiae abrotani herba* after hydrolysis.

Compound	Retention Time (min)	UV Detection	MS Detection	Concentration (µg∙mL^−1^)
Gentisic acid	2.15	NO	YES	NF
Caffeic acid	5.6	NO	YES	NF
Chlorogenic acid	5.6	NO	YES	NF
*p*-cumaric acid	8.7	YES	YES	3.63
Ferulic acid	12.2	YES	YES	27.7
Sinapic acid	14.3	YES	YES	79.95
Quercetol	26.8	YES	YES	33.31
Patuletin	28.7	YES	YES	19.04
Luteolin	29.1	YES	YES	1.29
Kaempferol	31.6	YES	YES	4.19

Notes: NF-not found, below limit of detection; Values are the mean ± SD (0.2%–1.5%) (*n* = 3); SD = standard deviation.

### 2.2. Total Polyphenol, Flavonoid and Hydroxycinnamic Derivatives Content

**Figure 2:**
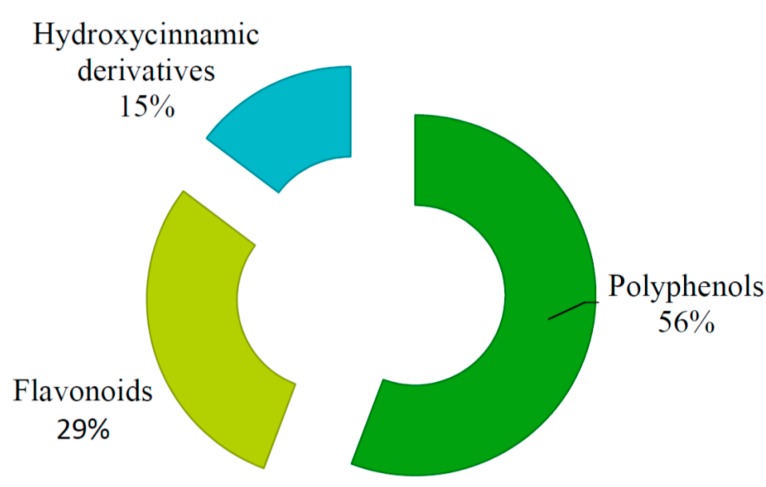
Content of polyphenols, flavonoids and hydrocinnamic derivatives in *Artemisiae abrotani herba*.

We analyzed the total content of polyphenols, flavonoids and hydroxycinnamic derivatives. Results can be found in Table 3 and Figure 2. The total polyphenol content is expressed in gallic acid equivalents (mg GAE/g plant material), the total flavonoid content is expressed in rutin equivalents (mg rutin/g plant material), and the total hydroxycinnamic derivatives content is expressed in caffeic acid equivalents (mg caffeic acid/g plant material).

**Table 3:** Total content of polyphenols, flavonoids and hydrocinnamic derivatives in *Artemisiae abrotani* *herba*.

Total Content of	Polyphenols (mg gallic acid/g HP)	Flavonoids (mg rutin/g HP)	Hydroxycinnamic Derivatives (mg caffeic acid/g HP)
*Artemisiae abrotani herba*	12.7	6.74	3.35
SD	0.44	0.32	0.06

HP = herbal product, SD = standard deviation.

### 2.3. Antioxidant Activity Assay

The antioxidant activity of the ethanolic extract of *A. abrotanum* L. was evaluated using the DPPH (di-(phenyl)-(2,4,6-trinitrophenyl) iminoazanium) method. We used ascorbic acid as positive control. A curve of % DPPH scavenging capacity *vs.* concentration was plotted and we calculated the IC_50_ value. IC_50_ is the concentration of a compound at which the response is reduced by half. The lower the IC_50_ value, the higher the antioxidant potential of the extract.

The results are presented in Table 4.

**Table 4:** DPPH free radical scavenging activity.

Product	IC_50_ (µg∙mL^−1^)	SD
Ascorbic acid	17.34	0.43
*Artemisiae abrotani herba* extract	284.5	16.21

IC_50_ = inhibitory concentration 50%; SD = standard deviation.

*A. abrotanum* L*.* received less research attention because it only contains traces of artemisinins [21,22]. However, the plant is rich in essential oil and several studies analyzed its composition and indicated possible applications [10,23]. Literature is scarce on information regarding the total herbal extract of *A. abrotanum* L., with little data about the polyphenolic profile distribution. After extensive research in several scientific databases (Medline, Web-of-Science, Science Direct, Springer Link, EbscoHost, Dr. Duke’s Phytochemical Database), we found indications that it contains polyphenols such as caffeic acid, chlorogenic acid, rutin and quercetin isomers [24,25].

This is the first in depth study to investigate the polyphenol profile of *A. abrotanum* L. Most polyphenols, hydroxycinnamic derivatives and flavonoids were identified and quantified for the first time in this study. The results indicate a unique distribution of these compounds, with higher levels of sinapic acid, rutin and patuletin.

In the herbal extract of *A. abrotanum* L., we identified several phenolic acids: gentisic, caffeic, chlorogenic, *p*-coumaric, ferulic and sinapic acid, both before and after acid hydrolysis. The quantitative analysis was possible for the *p*-coumaric acid, ferulic acid and sinapic acid and indicates sinapic acid as the main polyphenolic acid present in the herbal extracts, with a concentration of 34.56 µg∙g^−1^ before hydrolysis and 79.95 µg∙g^−1^ after hydrolysis. The differences before and after hydrolysis are probably due to the presence of complex superior esters which, after hydrolysis, liberate sinapic acid. Frequently, the concentration of some polyphenolic acids increases after hydrolysis [26,27].

We identified several flavonoid aglycones: quercetrol, luteolin, apigenin, and identified the most abundant flavonoids: hyperoside, rutin (highest concentration: 62.9 µg∙g^−1^), quercetol, patuletin (highest concentration: 19.04 µg∙g^−1^), luteolin, kaempferol.

In an *in vivo* test on mice and rats, Karakida *et al*. indicate sinapic acid as a possible candidate for a cerebral protective and cognition-improving drug. They showed that sinapic acid might protect against cerebral neuronal damage and death by inhibiting cerebral hypoxia [28]. Pari *et al*. proved that sinapic acid has a protective effect over arsenic induced toxicity in rats [29].

Rutin is used therapeutically for various diseases. It protects the brain against several insults through its antioxidant and anti-inflammatory properties. A study on rats with permanent bilateral carotid artery occlusion (BCCAO), a well-established model of chronic cerebral hypoperfusion, showed that rutin improves marked cognitive deficits and that it alleviates central cholinergic dysfunction, oxidative damage, inflammatory responses and neuronal damage in the cerebral cortex and hippocampus [30]. Direct evidence points to rutin as a therapeutically active molecule in the prevention and treatment of inflammatory bowel disease and colorectal carcinogenesis via attenuation of pro-inflammatory cytokine production [31]. Similarly, rutin protects against gastrointestinal adverse effects through the mechanism of inhibiting neutrophil infiltration, diminishing reactive oxygen species production and restoring the nitrite/nitrate balance [32]. Patuletin is a rare flavonoid with very few known sources in plant kingdom. Research is scarce on the subject, yet further investigations might reveal interesting biological activities [33].

The original distribution of polyphenols in *A. abrotanum* L. along with the discovery that it is a source of rare flavonoids opens the field for prospective investigations on new therapeutic uses for the medicinal plant.

Next, our results indicate that the plant contains significant amounts of polyphenols, flavonoids and hydroxycinnamic derivatives. Further research could improve the yield of extraction by optimizing the harvesting period (so that the plant reaches the maximum production of the secondary metabolites), the processing steps and the extraction protocol [34,35].

Polyphenols have been shown to have important biological activity in different illnesses [36]. To date there are more than 10,000 polyphenols identified in natural sources. Many of these polyphenols have been identified with a spectra of medicinal proprieties in various diseases [15]. It is considered that phenolic compounds have good antioxidant proprieties by inhibiting reactive oxygen species (ROS) production thus protecting against oxidative stress [37,38,39]. For all these proprieties, polyphenolic compounds have been studied as potential compounds in treating chronic diseases such as cardiovascular diseases, cancer, diabetes and neurodegenerative diseases [36,40].

The evidence that *A. abrotanum* L. contains significant amounts of polyphenols, together with an original distribution of polyphenols, flavonoids (aglycones and glycosylates) and hydroxycinnamic derivatives, opens the possibility for new health-related applications for this herbal extract.

The results of the antioxidant activity assay are well correlated with previous findings in literature, that show polyphenols as important antioxidant compounds [36,41,42]. So far, we did not find other data on the antioxidant potential of *A. abrotanum* L. As stated previously, it is highly probable that an optimized extraction will not only increase the concentration of polyphenols, but could also increase the antioxidant activity of the ethanolic extract [35,43]. Several improved methods, such as microwave-assisted extraction, ultrasound-assisted extraction and subcritical water extraction can significantly reduce the use of unsafe organic solvents and increase the extraction yield [44]. A new debate is carried out on the topic of green extraction methods and, in the future, these could become a routine option for laboratories following good practice guidelines [45].

*A. abrotanum* L. is used in Romania culinary as an aromatic plant and also in traditional medicine. Our results indicate that with its significant amounts of polyphenols, flavonoids and hydroxycinnamic derivatives, *Artemisiae abrotani herba* is an important source of dietary polyphenols. Dietary polyphenols have beneficial effects on human health with their antioxidant, anti-inflammatory and anticancer properties. Therefore, the consumption of plant-derived polyphenol-rich fruits, vegetables and beverages, is recommended for a healthy lifestyle [46].

## 3. Experimental Section

### 3.1. Plant Material and Extraction Protocol

*A. abrotanum* L*.* was identified and harvested from Cluj County, Transylvania, Romania, at the beginning of September 2013, during the flowering period. It belongs to a garden culture of aromatic plants kindly gifted by Prof. Dr. Mircea Tamas. The branches were cut and dried at room temperature, in a protected environment, then the leaves were detached from the branches. Voucher specimens were deposited at the Department of Pharmaceutical Botany Herbarium of the Faculty of Pharmacy, “Iuliu Hațieganu” University of Medicine and, Cluj-Napoca, Romania.

The herbal material was dried at room temperature in the shade, and grinded to fine powder (300 µm) before the extraction procedures. The samples were weighted (20 g) and extracted with 200 mL of ethanol (70%), in an ultra-sonication bath (30 min, at 40 °C). After that, the samples were cooled down and centrifuged at 4500 rpm for 15 min, and the supernatant was recovered.

### 3.2. Chemicals and Instruments

The following standards were used for HPLC-MS analysis: chlorogenic acid, *p*-coumaric acid, caffeic acid, rutin, apigenin, quercetin, isoquercitrin, quercitrin, hyperoside, kaempferol, myricetol, fisetin (from Sigma, St. Louis, MO, USA), ferulic acid, sinapic acid, gentisic acid, gallic acid, patuletin, luteolin (from Roth, Karlsruhe, Germany), cichoric acid, caftaric acid (from Dalton, Toronto, ON, Canada). HPLC grade methanol, ethanol, analytical grade orthophosphoric acid, hydrochloric acid and Folin-Ciocalteu reagent were purchased from Merck (Darmstadt, Germany), DPPH (2,2-diphenyl-1-picrylhydrazyl), sodium molybdatedihydrate, sodium nitrite, sodium hydroxide, sodium carbonate, sodium acetate trihydrate and anhydrous aluminum chloride were purchased from Sigma-Aldrich (Steinheim, Germany). All spectrophotometric data were acquired using a Jasco V-530 UV-VIS spectrophotometer (Jasco International Co., Ltd., Tokyo, Japan).

### 3.3. HPLC/MS Analysis

The experiment was carried out using an Agilent 1100 HPLC Series system (Agilent, Santa Clara, California, CA, USA) equipped with degasser, binary gradient pump, column thermostat, autosampler and UV detector. The HPLC system was coupled with an Agilent 1100 mass spectrometer (LC/MSD Ion Trap VL). For the separation, a reverse-phase analytical column was employed (Zorbax SB-C18 100 × 3.0 mm i.d., 3.5 μm particle); the work temperature was 48 °C. The detection of the compounds was performed on both UV and MS mode. The UV detector was set at 330 nm until 17.5 min, then at 370 nm. The MS system operated using an electrospray ion source in negative mode. For interpretation of data, we used the ChemStation and DataAnalysis software from Agilent.

The mobile phase was a binary gradient prepared from methanol and solution of acetic acid 0.1% (*v*/*v*). The elution started with a linear gradient, beginning with 5% methanol and ending at 42% methanol, for a 35 min duration; then isocratic elution followed for the next 3 min with 42% methanol. The flow rate was 1 mL∙min^−1^ and the injection volume was 5 μL.

### 3.4. Polyphenol Profile (Qualitative and Quantitative Analysis)

The determination was performed using the external standards method and it was used to identify and quantify nineteen phenolic compounds: eight phenolic acids and eleven flavonoids. The method was already set up and its application was proved in several other papers. The quantification was made only if the retention time of the compound was as for a standard mixture of polyphenols and if the recorded MS spectra also matched those of standard [27,47].

The MS signal was used only for qualitative analysis based on specific mass spectra of each polyphenol. The mass spectrometry (MS) spectra obtained from a standard solution of polyphenols was cross-checked in a mass spectra library. Later, the MS traces/spectra of the analyzed samples were compared to spectra from library, which allows positive identification of compounds, based on spectral mach. The UV trace was used for quantification of identified compounds from MS detection. Using the chromatographic conditions described above, the polyphenols eluted in less than 35 min (Table 5).

Four polyphenols could not be quantified in current chromatographic conditions due to overlapping detection (caftaric acid with gentisic acid and caffeic acid with chlorogenic acid). However, all four compounds were selectively identified in MS detection (qualitative analysis) based on differences between their molecular mass and MS spectra. The detection limits were calculated as the minimal concentration that produces a reproductive peak with a signal-to-noise ratio greater than three. Quantitative determinations were performed using an external standard method. Calibration curves in the 0.5–50 μg mL^−1^ range with good linearity (*R*^2^ > 0.999) for a five point plot were used to determine the concentration of polyphenols in plant samples. The polyphenol profile in the *A. abrotanum* L. extract was analyzed before and after acid hydrolysis.

**Table 5:** Retention times (t_R_) for the investigated polyphenols.

Peak No.	Phenolic Compound	t_R_ ± SD (min)	Peak No.	Phenolic Compound	t_R_ ± SD (min)
1	Caftaric acid *	2.10 ± 0.06	10	Rutin	20.20 ± 0.15
2	Gentisic acid *	2.15 ± 0.07	11	Myricetin	20.70 ± 0.06
3	Caffeic acid *	5.60 ± 0.04	12	Fisetin	22.60 ± 0.15
4	Chlorogenic acid *	5.62 ± 0.05	13	Quercitrin	23.00 ± 0.13
5	*p-*coumaric acid	8.7 ± 0.08	14	Quercetol	26.80 ± 0.15
6	Ferulic acid	12.2 ± 0.10	15	Patuletin	28.70 ± 0.12
7	Sinapic acid	14.3 ± 0.10	16	Luteolin	29.10 ± 0.19
8	Hyperoside	18.60 ± 0.12	17	Kaempferol	31.60 ± 0.17
9	Isoquercitrin	19.60 ± 0.10	18	Apigenin	33.10 ± 0.15

* overlapping in UV detection, only qualitative analysis possible using MS detection; SD (standard deviation).

### 3.5. Total Content of Polyphenols, Flavonoids and Hydroxycinnamic Derivatives

The total polyphenol content was determined using the protocol described in the European Pharmacopoeia, determination of tannins in herbal drugs (2.8.14), using the Folin-Ciocalteu reagent [48]. The absorbance is determined at 760 nm, using a UV-VIS Jasco V-530 spectrophotometer. The total polyphenol content is calculated using the equation obtained from the calibration curve of a series of gallic acid standard points (*R*^2^ = 0.996). The results are expressed in gallic acid equivalents (mg gallic acid/g herbal product (HP)).

For the quantification of the total flavonoids, we used the method described in the Romanian Pharmacopoeia in the *Betulae folium* monograph (10th edition) [49]. It is a spectrophotometric method (430 nm) that uses aluminum chloride solution as a color reagent. The results are expressed in rutin (g rutin/g HP) after extrapolation using the calibration curve of rutin (*R*^2^ =0.993).

The hydroxycinnamic derivatives were determined using a spectrometric method using Arnows’ reagent as previously described in the European Pharmacopoeia (10th Edition 2008-*Melissae folium* monograph) [48]. The percentage of hydroxycinnamic derivatives, expressed as caffeic acid equivalents (CAE) in dry herbal product (mg CAE/g HP), was determined using the equation of the calibration curve of a series of caffeic acid standard points (*R*^2^ = 0.998).

### 3.6. In Vitro Antioxidant Activity Assay-DPPH Method

DPPH (di-(phenyl)-(2,4,6-trinitrophenyl) iminoazanium) free radical method is an antioxidant assay based on electron-transfer that produces a violet color in ethanol. The free radical, stable at room temperature is reduced in the presence of antioxidant molecules, shifting the color of the solution from violet to yellow. The free radical scavenging activity of the herbal extracts was measured in terms of radical scavenging ability.

In clean and labeled test tubes, 2 mL of DPPH solution was mixed with 2 mL of different concentrations of herbal extracts. The tubes were incubated at room temperature in the dark for 30 min and the absorbance was measured at 515 nm using UV-VIS Spectrometer. The scavenging activity of the herbal extract was calculated using the formula:

Scavenging activity (%) = [(A − B)/A] × 100

where A is absorbance of DPPH and B is the absorbance of DPPH and herbal extract combination. The percentage of DPPH consumption in was converted to ascorbic acid equivalents using a calibration curve (*R*^2^ = 0.985) of ascorbic acid standard solutions (2–100 µg∙mL^−1^).

## 4. Conclusions

Natural products hold a wealth of useful therapeutic agents that people have used since early history. The empirical knowledge of the medicinal substances and their toxic potential was passed by oral tradition and sometimes recorded in texts such as Materia Medica.

In the ethanolic extract of *Artemisiae abrotani herba,* we screened nineteen polyphenols and flavonoids using a quantitative and qualitative LC-MS method. We identified seven aglycones and nine glycosylated polyphenols. The value of this study consists in its novelty. It is for the first time these biologically active compounds were identified and quantified in *Artemisiae abrotani herba*. What is more, our analysis shows an original distribution of polyphenols and flavonoids, with high contents of sinapic acid, rutin, ferulic acid, luteolin and patuletin.

Our study adds new data on the chemical composition of *A. abrotanum* L. The plant is used in traditional medicine for upper respiratory tract conditions. The unveiling of its content of bioactive polyphenols can lead to new applications for the herbal extract. Polyphenols are documented to protect against diseases that are linked to oxidative stress, such as cancer, cardiovascular or neurodegenerative diseases. The mechanisms of action of these compounds aren’t elucidated, yet it is acknowledged that they have antioxidant and anti-inflammatory properties. It is important to analyze the composition of natural products containing polyphenols to be able to identify new sources of biologically active molecules. More studies need to be conducted in the evaluation of the biological activity of *A. abrotanum* L. and its unique polyphenol content in order to quantify the antioxidant activity of its constituents. Further investigation will allow the implementation of an evidence-based strategy to use *A. abrotanum* L. to its full therapeutic potential.

## References

[1] Ramalingum N., Mahomoodally M.F. (2014). The therapeutic potential of medicinal foods. Adv. Pharmacol. Sci..

[2] Schmidt B., Ribnicky D.M., Poulev A., Logendra S., Cefalu W.T., Raskin I. (2008). A natural history of botanical therapeutics. Metabolism.

[3] Petrovska B.B. (2012). Historical review of medicinal plants’ usage. Pharmacogn. Rev..

[4] Abad M.J., Bedoya L.M., Apaza L., Bermejo P. (2012). The artemisia l. Genus: A review of bioactive essential oils. Molecules.

[5] Krishna S., Bustamante L., Haynes R.K., Staines H.M. (2008). Artemisinins: Their growing importance in medicine. Trends Pharmacol. Sci..

[6] Ho W.E., Peh H.Y., Chan T.K., Wong W.S. (2014). Artemisinins: Pharmacological actions beyond anti-malarial. Pharmacol. Ther..

[7] Coleman P.G., Morel C., Shillcutt S., Goodman C., Mills A.J. (2004). A threshold analysis of the cost-effectiveness of artemisinin-based combination therapies in sub-Saharan Africa. Am. J. Trop. Med. Hyg..

[8] Committee on Herbal Medicinal Products (2008). Assessment Report on Artemisia absinthium L., Herba.

[9] Van der Kooy F., Sullivan S.E. (2013). The complexity of medicinal plants: The traditional artemisia annua formulation, current status and future perspectives. J. Ethnopharmacol..

[10] Remberg P.B., Björkb L., Hedner T., Sterner O. (2004). Characteristics, clinical effect profile and tolerability of a nasal spray preparation of *Artemisia abrotanum* L. for allergic rhinitis. Phytomedicine.

[11] Brodin K., Alahyar H., Hedner T., Sterner O., Faergemann J. (2007). *In vitro* activity of *Artemisia abrotanu**m* extracts against malassezia spp., candida albicans and staphylococcus aureus. Acta Derm. Venereol..

[12] Radu A., Tămaș M., Băncilă E. (1973). Cercetări asupra uleiului volatil de *Artemisia abrotanum* L.—Identificarea eucaliptolului. Farmacia.

[13] Manach C., Scalbert A., Morand C., Rémésy C., Jiménez L. (2004). Polyphenols: Food sources and bioavailability. Am. J. Clin. Nutr..

[14] Procházková D., Boušová I., Wilhelmová N. (2011). Antioxidant and prooxidant properties of flavonoids. Fitoterapia.

[15] Tsao R. (2010). Chemistry and biochemistry of dietary polyphenols. Nutrients.

[16] Sak K. (2014). Cytotoxicity of dietary flavonoids on different human cancer types. Pharmacogn. Rev..

[17] Kanadaswami C., Lee L.T., Lee P.H., Hwang J.J., Ke F.C., Huang Y.T., Lee M.T. (2005). The antitumor activities of flavonoids. In Vivo.

[18] Sergent T., Piront N., Meurice J., Toussaint O., Schneider Y.J. (2010). Anti-inflammatory effects of dietary phenolic compounds in an *in vitro* model of inflamed human intestinal epithelium. Chem. Biol. Interact..

[19] Morris M.E., Zhang S. (2006). Flavonoid-drug interactions: Effects of flavonoids on abc transporters. Life Sci..

[20] Manzoor M., Anwar F., Mahmood Z., Rashid U., Ashraf M. (2012). Variation in minerals, phenolics and antioxidant activity of peel and pulp of different varieties of peach (*Prunus persica* L.) fruit from Pakistan. Molecules.

[21] Willcox M. (2009). Artemisia species: From traditional medicines to modern antimalarials and back again. J. Altern. Complement. Med..

[22] Dr. Duke’s Phytochemical and Ethnobotanical Databases-Farmacy Query. http://www.ars-grin.gov/cgi-bin/duke/farmacy2.pl?118.

[23] Tunon H., Thorsell W., Mikiver A., Malander I. (2006). Arthropod repellency, especially tick (ixodes ricinus), exerted by extract from *Artemisia abrotanum* and essential oil from flowers of dianthus caryophyllum. Fitoterapia.

[24] Chemicals in: *Artemisia abrotanum* L. (asteraceae). http://www.ars-grin.gov/cgi-bin/duke/farmacy2.pl.

[25] Spice pages: Southernwood (*Artemisia abrotanum*). http://gernot-katzers-spice-pages.com/engl/Arte_abr.html.

[26] Parvu M., Toiu A., Vlase L., Alina Parvu E. (2010). Determination of some polyphenolic compounds from allium species by HPLC-UV-MS. Nat. Prod. Res..

[27] Vlase L., Parvu M., Parvu E.A., Toiu A. (2012). Chemical constituents of three allium species from Romania. Molecules.

[28] Karakida F., Ikeya Y., Tsunakawa M., Yamaguchi T., Ikarashi Y., Takeda S., Aburada M. (2007). Cerebral protective and cognition-improving effects of sinapic acid in rodents. Biol. Pharm. Bull..

[29] Pari L., Mohamed Jalaludeen A. (2011). Protective role of sinapic acid against arsenic: Induced toxicity in rats. Chem. Biol. Interact..

[30] Qu J., Zhou Q., Du Y., Zhang W., Bai M., Zhang Z., Xi Y., Li Z., Miao J. (2014). Rutin protects against cognitive deficits and brain damage in rats with chronic cerebral hypoperfusion. Br. J. Pharmacol..

[31] Kwon K.H., Murakami A., Tanaka T., Ohigashi H. (2005). Dietary rutin, but not its aglycone quercetin, ameliorates dextran sulfate sodium-induced experimental colitis in mice: Attenuation of pro-inflammatory gene expression. Biochem. Pharmacol..

[32] Abdel-Raheem I.T. (2010). Gastroprotective effect of rutin against indomethacin-induced ulcers in rats. Basic Clin. Pharmacol. Toxicol..

[33] EMBL-EBI website Patuletin (chebi: 75164). http://www.ebi.ac.uk/chebi/searchId.do?chebiId=CHEBI%3A75164.

[34] Jun X. (2013). High-pressure processing as emergent technology for the extraction of bioactive ingredients from plant materials. Crit. Rev. Food Sci. Nutr..

[35] Ajila C.M., Brar S.K., Verma M., Tyagi R.D., Godbout S., Valéro J.R. (2011). Extraction and analysis of polyphenols: Recent trends. Crit. Rev. Biotechnol..

[36] Li A.N., Li S., Zhang Y.J., Xu X.R., Chen Y.M., Li H.B. (2014). Resources and biological activities of natural polyphenols. Nutrients.

[37] Poljsak B., Šuput D., Milisav I. (2013). Achieving the balance between ros and antioxidants: When to use the synthetic antioxidants. Oxid. Med. Cell. Longev..

[38] Halliwell B., Gutteridge J.M. (1995). The definition and measurement of antioxidants in biological systems. Free Radic. Biol. Med..

[39] Vauzour D., Rodriguez-Mateos A., Corona G., Oruna-Concha M.J., Spencer J.P.E. (2010). Polyphenols and human health: Prevention of disease and mechanisms of action. Nutrients.

[40] Finkel T., Holbrook N.J. (2000). Oxidants, oxidative stress and the biology of ageing. Nature.

[41] Scheepens A., Tan K., Paxton J.W. (2010). Improving the oral bioavailability of beneficial polyphenols through designed synergies. Genes. Nutr..

[42] Benzie I.F., Choi S.W. (2014). Antioxidants in food: Content, measurement, significance, action, cautions, caveats, and research needs. Adv. Food Nutr. Res..

[43] Benzie I.F.F., Wachtel-Galor S. (2011). Herbal Medicine.

[44] Xia E.Q., Deng G.F., Guo Y.J., Li H.B. (2010). Biological activities of polyphenols from grapes. Int. J. Mol. Sci..

[45] Chemat F., Abert Vian M., Cravotto G. (2012). Green extraction of natural products: Concept and principles. Int. J. Mol. Sci..

[46] Han X., Shen T., Lou H. (2007). Dietary polyphenols and their biological significance. Int. J. Mol. Sci..

[47] Mocan A., Crisan G., Vlase L., Crisan O., Vodnar D.C., Raita O., Gheldiu A.M., Toiu A., Oprean R., Tilea I. (2014). Comparative studies on polyphenolic composition, antioxidant and antimicrobial activities of schisandra chinensis leaves and fruits. Molecules.

[48] Council of Europe (2004). European Pharmacopoeia 5.0.

[49] Romanian Pharmacopoeia Commission National Medicines Agency (1993). Romanian Pharmacopoeia.

